# Images of bodies in mass and social media and body dissatisfaction: The role of internalization and self-discrepancy

**DOI:** 10.3389/fpsyg.2022.1009792

**Published:** 2022-12-20

**Authors:** Michelle Möri, Federica Mongillo, Andreas Fahr

**Affiliations:** Department of Communication and Media Research, University of Fribourg, Fribourg, Switzerland

**Keywords:** body images in media, body dissatisfaction, body shape scale, self-discrepancy theory, internalization of body ideals, survey design

## Abstract

**Introduction:**

The study examines the influence on body dissatisfaction of viewed images of bodies transmitted over mass media and social media, as mediated by the internalization of body ideals through media and self-discrepancy (the difference between the perceived actual self and the perceived ideal self).

**Method:**

In this study, the images of bodies individuals view in their everyday media diet are estimated using a newly developed pictorial scale for women (thinness) and men (muscularity). For participants, the perceived body image is formed through mass media (magazines, TV) and social media (Facebook, YouTube, Instagram, and Snapchat). The self-discrepancy theory is then used to explain the effect of images of bodies in the media on the internalization of these body ideals and body dissatisfaction.

**Results:**

Results show that Facebook and YouTube shape body ideals perceived to be prevalent in the media, negatively influencing internalization and self-discrepancy. Self-discrepancy, in turn, increases body dissatisfaction. However, for males, the perceived body ideals in the media did not affect body dissatisfaction, internalization, or self-discrepancy.

**Discussion:**

These results emphasize the importance of combining and comparing mass and social media and differentiating between female and male concerns regarding body image.

## Introduction

Social, political, and economic pressures establish and perpetuate notions and norms concerning body image, often equating thinness for women and muscularity for men with attractiveness (Bessenoff and Snow, 2006; Shen et al., 2022). Representations of bodies are present in people’s everyday lives. Still, with the rise of mass media and social media, people seem to have become obsessed with images of bodies or appearance (Groesz et al., 2002; Wykes and Gunter, 2005). Media can set and reinforce standards (e.g., the “ideal” body); provide models for social comparison; and eventually lead to psychological (e.g., body dissatisfaction), motivational (e.g., pressure to be thin or muscular), or behavioral outcomes (e.g., disordered eating, exercising, dressing; Diedrichs et al., 2011; Eyal and Te’eni-Harari, 2013). In media outlets, audiences are confronted with physically attractive models and appearance-focused content (de Vries et al., 2016). In particular, social media has developed significant power in promoting attractiveness, exercise, health, and well-being, upholding appearance ideals (Boepple et al., 2016; Griffiths et al., 2018), and increasing users’ weight concerns and body dissatisfaction (Stice and Shaw, 2002; Harper and Tiggemann, 2008; Kleemans et al., 2018).

Most of the studies analyzing the association between media and body image examine the influence of the amount of media use, for example, by asking about the daily hours spent on Instagram or watching television (e.g., Marengo et al., 2018; Vuong et al., 2021; Marques et al., 2022). Other studies manipulate the media content experimentally (McLean et al., 2016; Tiggemann et al., 2018; Anixiadis et al., 2019). Thereby, participants’ effective contact with images of bodies they encounter in their daily media consumption is not considered. While some studies compare the effects of different types of social media (Cohen et al., 2017; Alberga et al., 2018; Griffiths et al., 2018; Marengo et al., 2018) or different mass media outlets (Tiggemann, 2003), there is a lack of studies directly comparing the effects of other mass media outlets on social media outlets (Tiggemann, 2003). Thus, the primary goal of this study is to expand knowledge about the impact of the actual images of bodies transmitted by media by collecting data about those images people confront in their individual diets of mass media and different social media platforms. This adds to the existing research, as not only a general influence of media use is analyzed. More specifically, the images of bodies individuals perceive to be present in their media diet are examined.

When investigating the effect of media use or media-transmitted images of bodies on dissatisfaction, it is essential to consider the mechanism between media contact and expected outcomes. One approach that describes this connection is the self-discrepancy theory, which is often used in studies on body image (Forston and Stanton, 1992; Szymanski and Cash, 1995; Jung et al., 2001; Lantz et al., 2018; Flynn et al., 2020). The self-discrepancy theory posits that a discrepancy between the actual body image (how an individual believes their body looks) and the ideal body image (how an individual wants their body to look) can lead to adverse outcomes (Higgins, 1987). The permanent visibility of images of bodies in the media can influence the perception of an ideal body image shaped through the internalization of these promoted images of bodies as body ideals (Anixiadis et al., 2019). Thus, this study analyzes the role of internalizing body ideals and self-discrepancy through media consumption.

Even though males and females are both influenced by media, the ideal body standards differ, and so does the body image dissatisfaction between genders (Cohane and Pope, 2001). Whereas women in Western societies mainly focus on thinness (e.g., Bessenoff, 2006; Tiggemann and Slater, 2013; Lang et al., 2019), men commonly indicate a desire to develop and maintain muscularity (e.g., Scott and Donald, 2004; Frederick et al., 2007; Stratton et al., 2015; Flynn et al., 2020). Therefore, it is essential to address men and women differently when assessing the effects of media images of bodies (Flynn et al., 2020).

This study focuses on three perspectives regarding the relationship between media use and body dissatisfaction. First, we are interested in the body standards viewers perceive to be prevalent in the media that comprise their particular media diet, respecting individual differences in media consumption. Thereby, perceived body ideals will be measured in several traditional mass media (e.g., TV, magazines) and social media outlets (e.g., Facebook, YouTube, Snapchat, Instagram) to consider more than just one of the media sources shaping body standards. Second, the internalization of these body ideals and the perceived discrepancy between the actual and ideal self are introduced as mediators in the association between viewed media images of bodies and body dissatisfaction. Third, differences between women’s and men’s body ideals are considered, focusing on women’s drive for thinness and men’s drive for muscularity.

### Media exposure and body image

Body image is a multifaceted phenomenon that covers an individual’s attitude, feelings, and perceptions about their body size, body shape, and esthetic feelings (Cash, 2004; Wykes and Gunter, 2005). Regular exposure to pronounced thin or muscular bodies depicted in the media can make those bodies desirable. People see those depictions as standards to aspire to Bessenoff (2006). When analyzing the influence of images of bodies in media outlets, most studies ask about participants’ general use of media—for example, how often they read particular magazines (Tiggemann, 2003; Tiggemann and Miller, 2010), watch TV in general (Harrison, 2001), watch specific shows on TV (Hefner et al., 2014), or browse Instagram’s public content (Stein et al., 2019). Those measures can indirectly indicate which kinds of body images the participants are confronted with in their individual media diet.

Other studies investigate the general media use, and specific activities participants engage with on social media. This includes editing editing selfies before posting (McLean et al., 2015; Lonergan et al., 2019; Modica, 2020), the frequency of taking selfies (Modica, 2020) or posting selfies (Ridgway and Clayton, 2016), or the feedback individuals get on their posted pictures (Butkowski et al., 2019) and their influence on body satisfaction, body surveillance, or mood. These studies account for individual media use but mostly focus on the participants’ content, not the other images of bodies they might be confronted with.

In contrast, experimental studies use curated media content depicting various body images and measuring the effect before and after exposure. Some of these studies address only internal validity (Myers and Biocca, 1992; Heinberg and Thompson, 1995; Agliata and Tantleff-Dunn, 2004; Dittmar and Howard, 2004); more recent studies also include a good external validity in these experimental settings (Brown and Tiggemann, 2016; Casale et al., 2021; Sumter et al., 2022). Accurate individual media use is of minor importance in research designs that focus on the effects of particular content. At best, media use, media awareness, or media literacy are integrated into the model as a controlling factor (Anixiadis et al., 2019). Thus, the first goal of this study is to measure the perception of body standards typically viewed by media users in their *individual media diet*, consisting of traditional mass media and social media platforms, to ensure a high external validity. Thus, the first research question is:

*RQ1: Which media types shape the users’ perceived body image in their individual media diets?*

Media content analyses have frequently shown that the body type most commonly represented in the media does not match the entire population’s range of body shapes (Eyal and Te’eni-Harari, 2013). The media presents different body image cultures to men and women. For example, the media encourages women to control their weight and get thinner, mainly proliferating a *culture of thinness*. The media influence males, besides being thin also, to mold their bodies through exercise, proliferating a *culture of muscularity* (Agliata and Tantleff-Dunn, 2004; Scott and Donald, 2004; Holland and Tiggemann, 2016; Boursier and Gioia, 2022).^1^ Studies have shown the adverse effects of body ideals transmitted through mass media and social media on audiences’ body dissatisfaction (Grabe et al., 2008; Ferguson, 2013; Holland and Tiggemann, 2016; Mingoia et al., 2017; Huang et al., 2021).

Media exposure to desirable body shapes negatively influences one’s body image (Holmstrom, 2004; Boursier and Gioia, 2022; Shen et al., 2022), eating behaviors (Stice and Shaw, 2002; Griffiths et al., 2018; Saunders and Eaton, 2018; Guo et al., 2022), mental health (Kim and Sundar, 2012; Marengo et al., 2018; McCrory et al., 2022), and body satisfaction (Groesz et al., 2002; Grabe et al., 2008; Ferguson, 2013; Burnette et al., 2017; Wilhelm et al., 2019; Vuong et al., 2021). This negative effect on body satisfaction was shown in mass media, like television (Heinberg and Thompson, 1995; Hefner et al., 2014; Te’eni-Harari and Eyal, 2015) or magazines (Cusumano and Thompson, 1997; Tiggemann, 2003), and over various social media platforms including Facebook (Tiggemann and Slater, 2013; Meier and Gray, 2014; Griffiths et al., 2018), Instagram (Kleemans et al., 2018; Marengo et al., 2018; Tiggemann et al., 2018), Pinterest (Lewallen and Behm-Morawitz, 2016; Simpson and Mazzeo, 2017), and Snapchat (Marengo et al., 2018; Saunders and Eaton, 2018). Thus, as a first step, we assume that idealized images of bodies in the media (thinness for women, muscularity for men) lead to greater dissatisfaction with one’s body. In this study, this association will be further explored by combining mass media and social media to analyze whether different patterns emerge. Also, the hypothesis can be tested without experimentally manipulating thin/muscular body images. High external validity enables the analysis of this well-established relationship considering everyday media use.

*H1: The thinner or more muscular the viewers perceive the body ideal in the media, the stronger their body dissatisfaction.*

### Body ideals and internalization

Besides the direct effect of media exposure on body satisfaction, other factors can influence this relationship (Bessenoff, 2006). For example, the ideal thin-internalization describes the acceptance of social standards of thinness (Heinberg and Thompson, 1995; Cusumano and Thompson, 1997). This refers to a process described in the Tripartite Influence Model, which considers three main factors as sources of body dissatisfaction (Thompson et al., 1999; Keery et al., 2004b; Shroff and Thompson, 2006). Besides media, the other two factors are peers and parents. These three factors potentially influence body dissatisfaction and eating disturbances. However, they also indirectly lead to an internalization of societal appearance standards and comparison processes, shaping body dissatisfaction. The model describes, thus, sociocultural sources which can influence individuals and lead them to a negative evaluation of their bodies (Keery et al., 2004b; Shroff and Thompson, 2006). In this study, the focus lies on the media as one of these three possible influences on body dissatisfaction directly or indirectly through internalizing societal standards.

Media users can internalize body ideals they perceive to be prevalent in mass and social media. This can lead to greater body dissatisfaction when they cannot meet the typically unrealistic body ideals (Lavender et al., 2017; Vuong et al., 2021). Meta-studies have shown that media exposure, in general, can reinforce the internalization of body ideals for both genders and many ages (Huang et al., 2021; Paterna et al., 2021). The images of bodies perceived in the media can become personal standards of attractiveness (Thompson and Stice, 2001). Individuals try to attain internalized body ideals by monitoring their body appearance (Moradi and Huang, 2008; Moradi, 2010). Body depictions can strongly shape the internalized societal standards portrayed in the media (Holland and Tiggemann, 2016; Tatangelo and Ricciardelli, 2017; Rousseau and Eggermont, 2018). Through media internalization, individuals embrace media-promoted body ideals as personal body ideals, manifesting an ideal self and increasing body dissatisfaction (Durkin et al., 2007; Rodgers et al., 2014). Based on these studies, we test this association while considering the individuals’ perceived images of bodies in their everyday media diet and combining mass and social media outlets. As males and females process body images depicted in the media differently (Watson et al., 2019), we strive to test this widely known assumption for males and females separately.

*H1a: The effect of thin or muscular body depictions perceived in the media on body dissatisfaction is mediated through internalization of body ideals.*

### Body ideals and self-discrepancy

Besides increasing body dissatisfaction directly, internalizing body standards can emphasize the discrepancy between actual and ideal selves. Three domains of the self (actual, ideal, and ought) and two standpoints regarding the self (own and significant others) are distinguished in self-discrepancy theory. The actual self is a reflection of the beliefs about one’s attributes; the ideal self represents characteristics that one desires (Higgins, 1987), and the ought self represents details that one believes one should have (Heron and Smyth, 2013). People are motivated to establish consistency between the current self and the relevant self-guide, which in this context are the internalized ideals about body image (Higgins, 1987). Dissatisfaction increases when there is a discrepancy between the actual self and the ideal self (Higgins et al., 1986; Higgins, 1987; Strauman and Higgins, 1987). Although people differ in the degree to which they are motivated, they strive to reduce discrepancies by making their actual self match their ideal self (Heron and Smyth, 2013).

Previous studies have reported different variables that have mediating effects on self-discrepancy, such as self-esteem preventing negative body image after exposure to media images (Posavac et al., 1998; Dittmar and Howard, 2004), negative body image leading to eating disturbances (Forston and Stanton, 1992), and media images of bodies leading to body dissatisfaction (Harrison, 2001; Bessenoff, 2006). Thus, self-discrepancy can be introduced as a mediator between media body standards and body dissatisfaction. Again, this study tests whether this assumption applies to perceived images of the body in everyday media use. Comparing social media and mass media can give further insights into the connection between body images, body dissatisfaction, and self-discrepancy.

*H1b: The effect of thin or muscular body images perceived in the media on body dissatisfaction is mediated through self-discrepancy.*

### Research model

**FIGURE 1:**
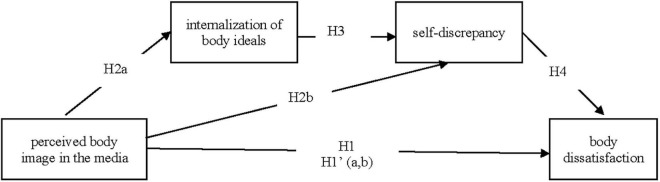
Research model of the influence of perceived body images in the media on body dissatisfaction, mediated through internalization, and self-discrepancy.

The media can play an essential role in shaping body ideals as viewers internalize body ideals that emphasize the discrepancy between the actual and ideal self. Based on the theoretical assumptions of the self-discrepancy theory and the body of the literature, the final research model (Figure 1) employs internalization and self-discrepancy as mediators between perceived body ideals in the media and body dissatisfaction. The directed hypotheses of the model are:

*H2: The thinner or more muscular the viewers perceive the body in the media image, the stronger (a) their internalization of body ideals and (b) their self-discrepancy.*

*H3: The more the viewers internalize the media’s body ideals, the stronger their self-discrepancy.*

*H4: The stronger the viewers’ self-discrepancy, the stronger their body dissatisfaction.*

## Materials and methods

### Research design and procedure

An online questionnaire was administered to test the research model. To survey a broad demographic, a quota sample from the UK was recruited *via Respondi*. Following the ethical regulations, only participants over 18 years old were eligible to participate. There was no upper age limit. The participants are members of the research panel *Respondi* and receive a small monetary incentive for each survey they complete. The sample was not representative but balanced between males and females and covered a broad range of ages and educational backgrounds. Nevertheless, the results cannot be generalized.

The panel members received an email invitation to the survey without any information about the topic. When clicking on the invitation link, they were informed on the first page that the study would be about their use of different media and body images. Also provided was an estimated time to complete the survey and contact information in case of questions or technical problems. Before starting the study, participants received written informed consent. They were informed about the data collection, the use and anonymization of their data, and their rights to withdraw from the study at any time.^2^ Only participants who accepted the informed consent could continue with the survey.

Participants then answered questions about their general media use and perception of body ideals portrayed on social media and in mass media. They had to choose a picture of a body that best corresponded to their actual and ideal selves. Afterward, they were asked about their satisfaction with their bodies. The survey ended by collecting participants’ sociodemographic information.

### Participants

A total of 293 participants (Table 1) from the UK were recruited. Data collection took place between June 13 and June 25, 2019. Participants needed, on average, 5 min and 29 s to complete the survey. In the sample, 53% of the participants were male (*n* = 156), and a majority were over 55 years old (41%). Most participants were married (53%), and most were European/Caucasian (88%). Participants had an average body mass index of 27 (BMI; underweight < 18.5, obese ≥ 40; *M* = 27.35, *SD* = 6.51; *n* = 274).

**TABLE 1:** Socio-demographic characteristics of the sample.

	*n*	%
**Gender**		
Male	156	53
Female	137	47
**BMI**		
Underweight (<18.4)	14	5
Normal weight (18.5-24.9)	93	32
Overweight (25-29.9)	89	30
High obesity (>30)	75	26
Prefer not to answer	22	7
**Age**		
18–25	9	3
26–35	36	12
36–45	58	20
46–55	71	24
Older than 55	119	41
**Marital status**		
Single (never married)	68	23
Married	139	47
In a domestic partnership	29	11
Divorced	45	15
Widowed	7	2
Prefer not to answer	5	2
**School education**		
Less than a high school diploma	44	15
High school diploma or equivalent	125	43
Bachelor’s degree (e.g., BA, BS)	74	25
Master’s degree (e.g., MA, MS, MEd)	21	7
Doctorate (e.g., Ph.D., EdD)	3	1
Other qualifications	21	7
Prefer not to answer	5	2
**Ethnicity**		
European/Caucasian	260	89
African/African American	2	1
Asian/Pacific islander	12	4
Other	15	5
Prefer not to answer	4	1

Only 15% of the participants did not use any social media platform daily, but they use social media in general. Facebook was the most-used platform, with 49% using it for 1 h a day, followed by YouTube with 45%, Instagram with 32%, and a mere 16% using Snapchat daily. The use of mass media was more common than in social media use in the sample. Only 4% of the participants reported using mass media for less than an hour daily. Television was used between 2 and 5 h a day (59%), whereas printed media was used for 1 h a day (46%).

### Measures

#### Scale development for body ideals

**FIGURE 2:**
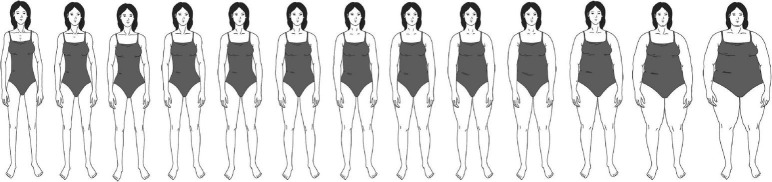
The newly developed body shape scale assesses women’s drive for thinness (1 = very thin, 13 = obese).

**FIGURE 3:**
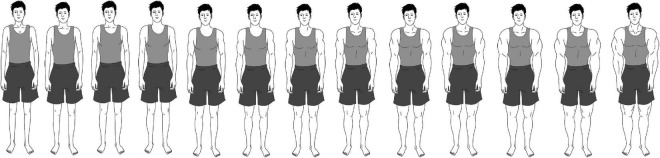
The newly developed body shape scale assesses men’s drive for muscularity (1 = leptosome, 13 = highly muscular).

Various techniques exist to measure the perception of body ideals, for example, linear, configurational, pictorial, or verbal (Wykes and Gunter, 2005). In this study, a configurational rating scale method was used. These measures have proven robust and valid (Thompson and Gray, 1995; Conti et al., 2013; Ralph-Nearman and Filik, 2018). The differences in body image, body ideal, and satisfaction for males and females had to be considered in building the scale. Existing scales for assessing body image either only account for one of the sexes without a corresponding equivalent for the other sex (e.g., male fit body scale; Ralph-Nearman and Filik, 2018), or the image scales only cover one of the ideals found in media (e.g., drive for thinness for males and females; Harris et al., 2008). Therefore, a new range of figures was created to capture two ideal body types often prevalent in media content. For females, the drive for thinness was chosen. For males, the drive for muscularity seems to be prevalent in different media outlets ranging from television to social media platforms, even though both drives can also be found for the other sex (e.g., Hildebrandt et al., 2004; Kelley et al., 2010). To capture females’ ideal for thinness, the scale includes 13 drawings of females that range from very thin to obese. For males, the scale consists of 13 corresponding pictures of males ranging from leptosome to overly muscular to capture males’ desire for muscularity (Figures 2, 3). To compare the scales for both sexes, the higher rankings corresponded more closely with the respective ideals (1 = *less thin/less muscular*, 13 = *thinner/more muscular*).

#### Actual self and ideal self

Using pictorial body scales to assess participants’ actual and ideal selves has proven valid in diverse settings and samples (e.g., Lynch et al., 2009; Conti et al., 2013; Ralph-Nearman and Filik, 2018). To measure the body image of the actual self, participants were asked to choose the picture from the body image scale of their gender that best matched their actual appearance (“What do you look like right now?”; *M* = 5.99, *SD* = 2.53). The ideal self of participants’ body image was assessed by asking the participants to choose the picture that best matched the appearance they wish to have (“What would you like to look like?”; *M* = 7.70, *SD* = 2.32).

#### The discrepancy between the actual self and the ideal self

The discrepancy indicator was calculated by subtracting the value of the actual self from the value of the ideal self for each participant as proven to be valid in previous research (e.g., Lynch et al., 2009; Conti et al., 2013). Therefore, positive scores indicate higher values for the ideal self than the actual self (72%), and zero indicates no difference between the actual and ideal self (20%). To better interpret the variable, participants with negative scores, meaning that their actual body was thinner/more muscular than the ideal self, were excluded (8%) so that a positive coefficient indicates an unhealthy discrepancy between the ideal and actual self.

#### Perceived body depictions in the media

In addition to the actual and ideal self, the pictorial body image scale was also used to assess the body depictions participants encountered in their individual media diets. Participants were asked to indicate the body depiction they are usually confronted with in their media diet (“Which of the following pictures correspond best to the men/women you see most frequently in the media you use?”). This was measured separately for television, magazines, YouTube, Facebook, Instagram, and Snapchat.

#### Internalization of body ideals

The thin/muscular bodies internalization was assessed using three items of the Sociocultural Internalization of Appearance Questionnaire (SIAQ) by Keery et al. (2004b). The scale is an extension of the validated Internalization Subscale of the Sociocultural Attitudes Towards Appearance Questionnaire-3 (Thompson et al., 2004). The scale was validated in samples from three countries and showed high internal consistency in six independent samples (Keery et al., 2004a). The items were adapted for females and males (“Looking at the body images in the media makes me want to lose weight/gain muscles”). *M* = 2.54, *SD* = 1.20.

#### Body dissatisfaction

For females, the Body Dissatisfaction Scale (Garner, 1991), a sub-scale of Garner’s Eating Disorder Inventory, was used (e.g., “I think my hips are too big”). This scale is a standardized clinical evaluation of symptomatology associated with eating disorders and is thus suitable for this study’s purpose. As an equivalent scale for males, the Body Dissatisfaction Scale (Yelland and Tiggemann, 2003) was used (e.g., “I think my biceps are too small”). This scale is based on the validated Body Esteem Scale (Franzoi and Shields, 1984) and has shown good validity in other studies (Yelland and Tiggemann, 2003). Five items out of the original nine from both scales were used. Only the statements that did not contradict one another were selected to still cover the concept as broadly as possible but with minimizing the number of items and effort of the participants (*M* = 2.97, *SD* = 1.24, α_females_ = 0.92, α_males_ = 0.77; *N* = 292; 5-point Likert-type scale from 1 = *never* to 5 = *always*).

### Statistical analysis

To test the impact of perceived media images on self-discrepancy and body dissatisfaction, we used partial least squares structural equation modeling (PLS-SEM). PLS is a causal-predictive approach to SEM that emphasizes prediction in estimating models derived based on theory and logic (Hair et al., 2019; Cho et al., 2022). PLS-SEM readily incorporates formatively specified constructs where the indicators are designed to jointly form said constructs (Sarstedt et al., 2016), which is the case for perceived body ideals in our model. Two path models were estimated based on the different body image scales (thinness for women and muscularity for men).

## Results

The results for men show that there was a negative effect of internalization on self-discrepancy (β = −0.39, *SD* = 0.19, *p* = 0.024). However, perceived media image had neither a direct nor an indirect (*via* self-discrepancy) effect on body dissatisfaction. As this study focuses on the media’s role in shaping the internalization of body ideals, self-discrepancy, and body dissatisfaction, only the model based on women is included below. The null results for men are taken up again in the discussion.

**FIGURE 4:**
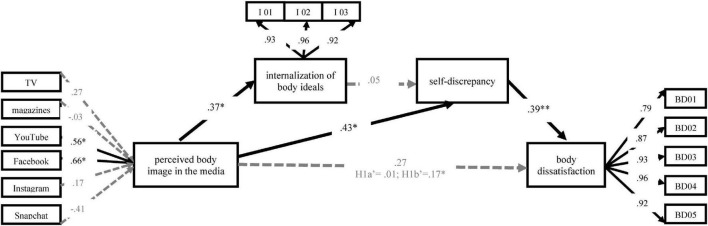
Research model for females and their drive for thinness. Indirect effect through internalization and self-discrepancy (H1a), indirect effect through self-discrepancy (H1b). *p < 0.05, ^**^p < 0.01.

The reflective measurement model results (see Figure 4 with further details in Tables 2–4) show that the construct measures exhibit sufficient internal consistency, reliability, and convergent validity (see Table 2). In the formative measurement model (see Table 3), we controlled for collinearity with the VIF values (Hair et al., 2016). In the next step, we ran bootstrapping (10,000 subsamples) to assess the significance of the formative indicator weights. Weights for Facebook and YouTube were significant. As the loadings of all media perceptions (besides magazines) on perceived body image were 0.50 or larger, these indicators made a decisive contribution to the construct. They were kept in the model based on theoretical assumptions. The indicator weights answered RQ1, which asked which media outlets shape the respondents’ body images. YouTube and Facebook were the essential factors forming the perceived body ideal in the media and thus created self-discrepancy and, eventually, body dissatisfaction.

**TABLE 2:** Reliabilities for reflective measured latent constructs.

	CE	SD	*t*	*P*	95% CI
**Average variance extracted**					
Body dissatisfaction	0.75	0.06	13.65	<0.001	[0.64, 0.82]
Internalization	0.87	0.04	21.00	<0.001	[0.81, 0.92]
**Composite reliability**					
Body dissatisfaction	0.94	0.02	46.59	<0.001	[0.90, 0.96]
Internalization	0.95	0.02	42.59	<0.001	[0.93, 0.97]
**Cronbach’s alpha**					
Body dissatisfaction	0.92	0.02	38.12	<0.001	[0.87, 0.95]
Internalization	0.93	0.02	42.19	<0.001	[0.88, 0.95]
**Rho**					
Body dissatisfaction	0.93	0.03	32.68	<0.001	[0.86, 0.95]
Internalization	0.93	0.35	2.65	0.004	[−7.34, 0.95]

AVE: Degree to which a latent construct explains the variance of its indicators (convergent validity; AVE > .5 for good fit). Composite Reliability: Measure of internal consistency reliability. Cronbach’s alpha: Measure of internal consistency reliability assuming equal indicator loadings (more conservative). Cronbach’s alpha is the lower bound, composite reliability the upper bound for internal consistency reliability. Rho: Measure for composite reliability, Rho > 0.7 for good fit.

**TABLE 3:** Outer weights of formative indicators for individuals’ body images in the media.

Outer weights	CE	SD	*t*	*P*	95% CI	VIF
TV	0.27	0.26	1.04	0.150	[−0.06, 0.76]	1.36
Magazines	−0.03	0.36	0.09	0.463	[−0.57, 0.61]	1.36
YouTube	0.56	0.27	2.10	0.018	[0.25, 1.01]	1.17
Facebook	0.66	0.39	1.70	0.044	[0.05, 1.20]	2.34
Instagram	0.17	0.47	0.36	0.358	[−0.53, 0.97]	2.61
Snapchat	−0.41	0.44	0.94	0.174	[−1.18, 0.21]	2.26

Outer weights: Result of the multiple regression indicates the relative importance in the formative measurement model. Higher values represent stronger relative importance of the indicator. VIF, variance inflation factor quantifies the severity of collinearity among the indicators. VIF should be lower than 3 for a good fit.

**TABLE 4:** Effects of body images in the media on body dissatisfaction mediated through self-discrepancy and internalization.

	CE	SD	*t*	*P*	95% CI
**Path coefficients**					
Internalization					
Body images in the media	0.37	0.20	1.82	0.035	[−0.62, −0.54]
Self-discrepancy					
Body images in the media	0.43	0.24	1.79	0.037	[−0.12, 0.70]
Internalization	0.05	0.24	0.20	0.419	[−0.34, 0.46]
Body dissatisfaction					
Body images in the media	0.27	0.18	1.54	0.061	[−0.68, 0.40]
Self-discrepancy	0.39	0.15	2.66	0.004	[0.18, 0.63]
**Specific indirect effects**					
Body images in the media → internalization → self-discrepancy	0.02	0.13	0.14	0.443	[−0.15, 0.27]
Body images in the media → self-discrepancy → body dissatisfaction	0.17	0.10	1.61	0.053	[0.06, 0.40]
Internalization → self-discrepancy → body dissatisfaction	0.02	0.10	0.20	0.420	[−0.08, 0.24]
Body images in the media → internalization → self-discrepancy → body dissatisfaction	0.01	0.05	0.14	0.446	[−0.04, 0.14]
**Total indirect effects**					
Internalization → body dissatisfaction	0.02	0.10	0.20	0.420	[−0.08, 0.24]
Body images in the media → body dissatisfaction	0.17	0.10	1.78	0.038	[0.06, 0.38]
Body images in the media → self-discrepancy	0.02	0.13	0.14	0.443	[−0.15, 0.27]
**Total effects**					
Internalization → body dissatisfaction	0.02	0.10	0.20	0.420	[−0.08, 0.24]
Internalization → self-discrepancy	0.05	0.24	0.20	0.419	[−0.34, 0.46]
Body images in the media → body dissatisfaction	0.44	0.14	3.22	0.001	[−0.58, 0.53]
Body images in the media → internalization	0.37	0.20	1.82	0.035	[−0.62, 0.54]
Body images in the media → self-discrepancy	0.44	0.19	2.36	0.009	[0.00, 0.64]
Self-discrepancy → body dissatisfaction	0.39	0.15	2.66	0.004	[0.18, 0.63]
**Adj. *R*^2^**					
Body dissatisfaction	0.28	0.12	2.27	0.012	[0.00, 0.38]
Internalization	0.11	0.14	0.79	0.215	[−0.03, 0.27]
Self-discrepancy	0.16	0.14	1.13	0.128	[−0.05, 0.33]

Model fits: saturated model SRMR = 0.07; Chi-Square = 126.84; NFI = 0.72. Estimated model: SRMR = 0.12; Chi-Square = 135.12; NFI = 0.70.

Overall, the model fit indices are relatively low (SRMR = 0.12, NFI = 0.70). In PLS models, the classical goodness-of-fit tests are not very informative, as they usually compare the covariance matrices of a saturated vs. an estimated model. With partial least square estimation, a formative indicator can be built, not referring to the covariance matrix. Literature on PLS also suggests not relying on these indices (Henseler and Sarstedt, 2013). Therefore, the indices are reported for transparency, but the models were not adopted data-driven to improve model fit with assumptions possibly violating the theoretical assumptions.

H1 proposed that the thinner the body depictions are perceived in the media, the higher the body dissatisfaction will be. However, the data showed no significant direct effect of perceived thinness on body dissatisfaction (β = 0.27; *SD* = 0.18, *p* = 0.061; H1 rejected). The thinner individuals perceived the body images in the media to be correlated with a higher internalization of body ideals through media (β = 0.37, *SD* = 0.20, *p* = 0.035, H2a confirmed). However, internalization showed no effect on self-discrepancy (β = 0.05, *SD* = 0.02, *p* = 0.419; H3 rejected). A more pronounced self-discrepancy was associated with a higher body dissatisfaction (β = 0.39, *SD* = 0.15, *p* = 0.004; H4 accepted). The thinner the body images in the media were perceived to be, corresponded with a higher discrepancy between the actual self and the ideal self (β = 0.43, *SD* = 0.24, *p* < 0.037; H2b confirmed). The thinner women perceived the body depictions on YouTube and Facebook, the more pronounced they judged the difference between their actual body image and the body shape they wished to have. While the direct effect of the perceived body ideals in the media on body dissatisfaction was not significant, the indirect effect through self-discrepancy was significant (β = 0.17, SD = 0.10, *p* = 0.038; H1b confirmed), but not through internalization and self-discrepancy (H1a rejected; see Table 4 for more details).

### Exploratory analyses

As in the PLS models, only participants with valid answers for all media outlets could be considered; a supplementary analysis was run to explore the hypotheses only for mass media. This model introduced TV and magazines as formative indicators of body image. The results show that, in particular, magazines (β = 0.71; *SD* = 0.34, *t* = 2.01, *p* = 0.018) and in tendency TV (β = 0.49; *SD* = 0.35, *p* = 0.083) form individuals’ media-perceived body ideal. Interestingly, this perceived body ideal formed from traditional mass media *directly* explains body dissatisfaction (β = 0.30; *SD* = 0.10, *t* = 2.872, *p* = 0.002), but the indirect effect *via* a pronounced self-discrepancy disappears (β = 0.05, *SD* = 0.07; *p* = 0.239). The effect of perceived body image on self-discrepancy is no longer significant (β = 0.09, *SD* = 0.13; *t* = 0.723, *p* = 0.235). This means that traditional media can still contribute to body dissatisfaction but not by triggering self-discrepancy beliefs.

## Discussion

The current study aimed to contribute to the research on the influence of media-transmitted body ideals in traditional mass and social media on body dissatisfaction. It sought to explain this effect through internalizing body ideals and perceived self-discrepancy between how one looks and wishes to look. Three relevant contributions of the study should be highlighted: First, the study used all participants’ perceived media-transmitted body ideals within the background of their actual media diet, including traditional mass media and several social media outlets. Second, this estimation took advantage of a specifically adapted image scale to assess the fragile construct of body image as intuitively as possible. Third, the measures were collected for males (muscularity) and females (thinness) within a comparatively broad sample of demographic structures.

For this study, a pictorial-figure rating scale was developed to measure females’ drive for thinness and males’ drive for muscularity. The use of a visual scale for participants to indicate their media perception maximizes the intuitiveness of the measurement. Both scales were applied in this study to measure the participants’ actual body shape, their ideal body shape, and the body shapes they are confronted with on social media and mass media. The 13 images of body shapes are a major contribution of this study to the existing literature, as pictorial rating scales have proven to be a valid and reliable way to measure body images (Thompson and Gray, 1995; Ralph-Nearman and Filik, 2018). The scale is based on an often validated and applied pictorial scale that only consists of seven figures (Stunkard et al., 1983). The newly developed scale adds intermediate figures to this scale, allowing a more nuanced measurement of body shapes. Nevertheless, the newly developed scale must be validated to fully ensure a valid and reliable measurement in future studies.

According to the self-discrepancy theory, the discrepancy between the actual and ideal body image can lead to adverse outcomes such as negative feelings or even depression (Higgins et al., 1986; Higgins, 1987). Thus, the first hypothesis was that the thinner or more muscular the viewers perceive the body ideal to be in the media, the stronger their body dissatisfaction should be. The data of this study showed that the thinner the body images in social media are perceived to be for women, the more pronounced the discrepancy between the actual self and the ideal self. This confirms that the thinner or more muscular the viewers perceive the body ideal in the media, the stronger their body dissatisfaction (H1) and the results of previous studies on this association (Harrison, 2001; Halliwell and Dittmar, 2006; Hefner et al., 2014) and underlines the importance of media in this context of body image concerns.

Through media internalization, individuals endorse media-promoted body ideals as standards of attractiveness to be attained, resulting in the manifestation of an ideal self (Bessenoff, 2006). The confrontation with body ideals in the media can lead to an unrealistic ideal self, usually manifesting as social media outlets being prone to increase the internalization of body ideals (Perloff, 2014; Mingoia et al., 2017). This assumption was tested: The effect of thin or muscular body depictions perceived in the media on body dissatisfaction is mediated through the internalization of body ideals. The results showed that the thinner body depictions in the media are perceived to be, the more robust the internalization of these ideals. However, this did not lead to a more pronounced discrepancy between the actual and ideal selves. More internalization of the media’s body ideals did not lead to a stronger self-discrepancy among viewers. This could be due to several reasons. Media internalization was considered a mediator, but it could also be argued that it is instead a trait and should be introduced as a moderator (Bessenoff, 2006; Krawczyk and Thompson, 2015; McLean et al., 2016; Vuong et al., 2021). Additionally, the association between internalization and body dissatisfaction can be explained through self-discrepancy and other processes such as body surveillance (Fitzsimmons-Craft et al., 2012), which should be considered in future studies.

The indirect effect of perceived body ideals in the media on body dissatisfaction *via* self-discrepancy indicates that the association between media perception and body dissatisfaction can partially be explained by triggering a pronounced gap between perceived body ideals and desired appearance. The effect of thin or muscular body images perceived in the media on body dissatisfaction is mediated through self-discrepancy. Besides media exposure, other drivers for the actual/ideal/ought self-congruency may be present. Moreover, the effect might be more pronounced if moderators introduced the association between media perceptions and self-discrepancy. These could be, for example, personality traits (Roberts and Good, 2010), motives for media use (Tiggemann, 2005), media literacy (Yamamiya et al., 2005; Levine and Murnen, 2009; Anixiadis et al., 2019), and parasocial relationships with media characters or character liking (Eyal and Te’eni-Harari, 2013; Te’eni-Harari and Eyal, 2015). It can also be argued that self-discrepancy and internalization of body ideals may drive or reinforce a particular media use or perception of media content (Rousseau and Eggermont, 2018; Marques et al., 2022). However, causality cannot be proven using our non-experimental cross-sectional design.

Although numerous studies investigate the association between body image and mass media or social media exposure, there is still a dearth of literature directly comparing the effects of traditional mass media to social media (Perloff, 2014). The strength of this study is that the perceived mediated body ideal was considered a formative construct, referring to an index of a weighted sum of variables of perceived body ideals in different media outlets. The data suggest that, in particular, YouTube and Facebook contribute to the media-transmitted body ideal for females, triggering self-discrepancy and eventual body dissatisfaction. Surprisingly, Instagram was not an important factor in this process, contradicting the results of other studies in which Instagram was a driver for self-discrepancy, body dissatisfaction, or other body image concerns (Tiggemann et al., 2018; Anixiadis et al., 2019; Stein et al., 2019; McCrory et al., 2022). Traditional mass media do not play a crucial role in influencing body ideals. Social media, with its pronounced active, selective, and highly individual choice of content, triggers social comparison and eventually leads to pronounced self-discrepancy (Marengo et al., 2018; McCrory et al., 2022).

The results for men and the media’s influence on their body dissatisfaction through body ideals with a drive for muscularity are discussed separately. For men, all media indicators had non-significant weights to form the latent variable perceived images of bodies in media. Thus, the different images of bodies perceived to be prevalent on these other media platforms do not seem to result in a general construct measuring body images in the media. Accordingly, no effects of these general body images can be found for self-discrepancy or body dissatisfaction. Studies investigating men’s body perceptions of their peer groups found that men are more likely to compare themselves to their peers than media depictions and that peers have more significant effects on body satisfaction (Yamamiya et al., 2005; Ferguson, 2013; Piatkowski et al., 2022).

There are several possible explanations for our results for men. First, this study only examined men’s drive for muscularity, which limits the findings. For men, there is a dual drive to be thin and to be muscular, and they may be split between both drives (Hildebrandt et al., 2004). Men were shown to have the desire to look lean with low body fat (e.g., Ricciardelli, 2012; Field et al., 2014), and the internalization of these thin ideals through media was shown to increase male body dissatisfaction (Dye, 2016b). It could be that rather than the drive for muscularity, a combination of both these drives for ideal body image or only the drive for thinness on the different media platforms influences men’s body dissatisfaction. The inclusion of only one of these body image desires does not cover men’s body ideals holistically and limits this study’s findings. Second, it could be that media probably influence men less, and they react differently to body ideals presented in media than females (Watson et al., 2019), as shown in other studies (Yamamiya et al., 2005; Ferguson, 2013; Piatkowski et al., 2022). Other factors, e.g., social comparison, could be more relevant regarding men’s body dissatisfaction. Also, the media content and other factors, for example, users’ comments, could influence men’s reactions to body images (Kim, 2021). Additionally, our sample consisted of men from different age groups, while many studies are conducted with relatively young samples. This raises the question if these findings are also applicable to the general population and would need to be tested with a representative sample.

The present study has some further limitations. First, the validity of measuring media perception could be criticized. As we measured the perceived body ideals in mass media and on social media, we knew neither the exact content participants were consuming nor the amount of the overall media consumption. Therefore, the actual body depictions presented in the media could not be verified, and the participants experienced the perceived body image subjectively. Another drawback is asking for only one body ideal as an indicator of all perceived body ideals in the media. It is a broad indicator (individual yet aggregated) and just functions as a point estimator for a wide variety of body shapes to which users are exposed in their media diet. Future studies might use the scale to ask people about a range of body images rather than forcing them to choose one picture. Furthermore, both the drive for thinness and the drive for muscularity should be examined for males and females (Kelley et al., 2010), as media could influence body ideals in more than just one way, and all individuals, regardless of their gender, can have a drive for thinness and/or muscularity (e.g., Dye, 2016a; Huang et al., 2021). In media, different body ideals can be represented for both gender (Huang et al., 2021), influencing their body dissatisfaction. Future studies should include these dual drives for thinness and muscularity for all individuals.

Second, social comparison theory was not explicitly considered. The theory establishes individuals’ need to compare themselves with others to determine their levels of abilities and success (Festinger, 1954). Many studies dealing with body image and media seek to explain the influence of media exposure on body dissatisfaction with upward social comparison processes between audiences and the people represented in the media (Field et al., 1999; Martin et al., 2004; Tiggemann and McGill, 2004; Bessenoff, 2006). In the research model of the present study, we included only the perceived body ideal in the media so no specific person would be available for social comparisons in the context of this ideal. However, social comparison theory could provide valuable insight into media influences on body dissatisfaction in future studies.

Third, the sample of the study limits the findings of this study. It was a non-representative sample, not allowing generalization of the results. However, a wide spectrum of age, BMI, and gender-balanced sample was achieved. The final sample in the analysis was relatively small, as only participants indicating body images for all kinds of media were considered. This was necessary for comparing the media outlets and the meaningful construction of the latent variable perceived body ideal. Although pairwise deletion would have resulted in different calculations, the analysis would have been based on different sample sizes, which can bias the results.

Further, the small sample size did not allow the integration of more variables into the model to, for example, control for age or BMI, as the model would have been too complex to estimate. This is a limitation to the findings, as BMI was shown to be a detrimental factor regarding body dissatisfaction (Conti et al., 2013; Marques et al., 2022). In the exploratory analysis, we further tested a model with only mass media, television, and magazines to enhance a larger sample. Thereby, mass media was shown to influence body dissatisfaction directly. This would mean that although social media is prevalent in forming body ideals, mass media still have power in this regard.

## Conclusion

The current study contributes to the discussion on the association between media exposure and body dissatisfaction as mediated by self-discrepancy and internalization. The study contributes to the existing literature by providing a newly developed pictorial scale with 13 images to measure females’ drive for thinness and males’ drive for muscularity. The scale was employed to measure the actual self, the ideal self, and the perceived body shapes in media. The body images individuals perceive to be present in the different media outlets could be considered. This ensures higher external validity than just examining the amount of time spent with certain media outlets. Data showed that the direct effects of perceived media-transmitted body ideals on body dissatisfaction for women are mediated through self-discrepancy but not internalization. Mainly, social media shape the body images perceived to be present for women. For men, the perceived body ideal for muscularity did not trigger internalization of body ideals, self-discrepancy, or body dissatisfaction. Future studies should continue to analyze the role of mass and social media and body image concerns while distinguishing between women’s and men’s transmitted body ideals.

## Data availability statement

The data presented in this study can be found in the online repository OSF: https://osf.io/gzafh/.

## Ethics statement

Ethical review and approval was not required for the study on human participants in accordance with the local legislation and institutional requirements. The patients/participants provided their written informed consent to participate in this study.

## Author contributions

AF and FM developed the research idea. FM performed the data collection. MM wrote the theoretical part and discussion, described the method, and conducted the data preparation. AF ran the statistical analysis and write-up, supervised the project, and performed the proofreading of the manuscript. All authors contributed to the article and approved the submitted version.

## Footnotes

1 Different body ideals exist for males. Besides the desire for increased muscularity, there is also the desire for weight change and reduced body fat (e.g., Drewnowski et al., 1995; Yelland and Tiggemann, 2003; Hildebrandt et al., 2004). However, in this study, only males’ drive for muscularity is analyzed.

2 The study was not approved by an ethics committee, as to the time of data collection the Institutional Review Board did not exist. The informed consent data collection procedure were in line with ethical guidelines and conform to the IRB directives of today.
